# Equipping future doctors: incorporating management and leadership into medical curriculums in the United Kingdom

**DOI:** 10.1007/s40037-017-0327-3

**Published:** 2017-02-15

**Authors:** Aniket Sonsale, Reshma Bharamgoudar

**Affiliations:** 1 Franklin-Wilkins Building, King’s College London, London, UK; 2 Imperial College London Business School, London, UK

**Keywords:** Leadership, Management, Curriculum, Medicine

## Abstract

Throughout their careers, doctors are likely to come across complex management and leadership scenarios that many would not have had prior training in. Expectations of doctors are rising and it is becoming increasingly necessary to be able to astutely handle a variety of situations. Medical curricula must reflect this change and adapt to include the teaching of key management and leadership skills. Despite budgeting pressures, the National Health Service continues to spend vast sums of money on external management consultants. The 2013 Francis Report stressed the need for better management skills and leadership, especially in doctors who were identified as the spearheads of change. This view is backed up by senior professionals who stress that by incorporating it into undergraduate curricula, doctors will be equipped with the skills to flourish in the future. The challenges of doing so must be highlighted, since the teaching of managerial and leadership concepts must effectively combine theoretical approaches with practical applications. Empowering students of today will enable them as tomorrow’s doctors to tackle the challenges of modern medicine

## Introduction

Leadership matters. Whether it is the junior doctor simultaneously handling four patients in the emergency room, the overwhelmed sole surgical registrar on call at 2am or the consultant liaising with colleagues to keep his department afloat; these doctors may not have had substantive prior training in dealing with these complex situations. Whilst doctors have the skills for even the most intricate clinical scenarios, it is the lack of leadership and management training that is most concerning. As fourth year medical students with a Bachelor’s in Management, we have been introduced to a multitude of skills that we feel will be invaluable to our future medical careers. With responsibilities of doctors rising and a developing litigation culture, it is increasingly important to take charge and astutely handle a variety of situations. In the past there was emphasis on the doctor as a transactional leader, yet recent changes in modern medicine increasingly place demands on the doctor to be a transformational leader; one that empowers peers and seeks to achieve collective organizational change [1]. This change is often termed ‘bottom-up’ and seeks to achieve greater professional involvement in key decision-making areas, as it is often the clinicians who are better able to determine the needs of their patients [2]. Such viewpoints were the main arguments for changing the healthcare delivery model in the UK as part of the 2012 Health and Social Care Act, which empowered frontline general practitioners in determining the best way to allocate scarce resources in the healthcare system [3]. Undergraduate medical curricula must therefore reflect this and adapt to include leadership and management to prepare students for the evolving nature of healthcare.

## Management and leadership in healthcare: what do they mean?

Management is defined as the ‘act or skill of controlling or making decisions’ [4]. Whereas ‘traditionally’ it may have been seen in predominantly business-focussed industries, it is equally important in the healthcare setting where it can be viewed as patient management, people management and organizational management. A seminal publication in medical leadership and management, the 2008 Darzi report, described empowered clinical professionals with strong clinical management skills as having a key role to play in raising standards in the National Health Service (NHS) [5]. Leadership is arguably equally important in today’s climate and it can be described as ‘the art of influencing others to their maximum performance to accomplish any task, objective or project’ [6, p. 9]. This is reflected in Martin and Learmonth’s examination health policy texts in the United Kingdom (UK), which highlighted a language shift from ‘management’ to ‘leadership’ in public policy discourse [7]. Furthermore, the highly influential CanMEDS (Canadian Physician Competency Framework), has switched its emphasis from ‘manager’ to ‘leader’ in the light of changes in the expectations of physician competencies [8].

## Importance of management and leadership

Some may feel that management is not the responsibility of a doctor. It is estimated that there are approximately 50,000 managers in the NHS, amounting to 4% of the workforce [9]. With 8% of the NHS budget spent on management and administration, it is arguable that there are already adequate numbers of managers employed by the NHS [10]. As such, they represent one-third of the total managerial workforce in the economy of the UK [9]. In spite of recent pressures on the NHS budget, the Government continues to spend a substantial amount of money on outsourcing management, despite a monitor report acknowledging that this expenditure is often ineffective [11, 12]. In 2014/2015, £420 million was spent on management consultants, and this represents an opportunity cost whereby this money could perhaps be used in other areas for greater benefit. As it is, doctors highly influence the budget spending in the NHS, therefore it is illogical that there remains a lack of participation of doctors in management roles [10]. Hence it will not only enhance care by encouraging doctors to take ownership over the NHS but it will undoubtedly shape the organization for the better.

One of the darkest periods in the history of the NHS was the Mid Staffordshire scandal. The subsequent Francis Report raised issues regarding mismanagement at senior level and highlighted that doctors were not at the ‘forefront of promoting change’ [13, p. 43]. This stressed the need for more training of leadership and management skills for staff. Such situations clearly must be avoided in the future and are a stark reminder of organization-wide mismanagement and leadership failure. The 2012 General Medical Council guidelines on Leadership and Management specifically refer to the responsibilities of a doctor extending beyond more than just ‘being a good clinician’ [14, p. 4]. Some of the highlighted roles include: ‘planning, using and managing resources’ and ‘providing leadership and vision to the organisation’ [14, p. 31, 4]. Such responsibilities are not restricted to UK clinicians, rather they are seen as a clinician’s responsibility in other healthcare systems around the world. CanMEDS is widely heralded as being influential in changing the way training occurs in countries worldwide since its inception in Canada in 1996 [8]. It has in fact influenced UK training, as well as that within countries such as Australia, the Netherlands and USA [15, 16]. CanMEDS clearly specifies that physicians must be able to ‘lead personnel’ and ‘apply evidence and management processes to achieve cost-appropriate care’ [8, p. 10]. These values appear to have filtered through successfully in the UK in the form of the Medical Leadership Competency Framework, devised to support the implementation of the General Medical Council Outcomes in Leadership and Management [17]. The Medical Leadership Competency Framework acknowledges the points made in CanMEDS and outlines a three-stage framework, at each level of a doctor’s training, to improve these skills within students and doctors. Furthermore, this was improved in 2013 with the introduction of the Healthcare Leadership Model, which is aimed at multiple levels within the NHS and encourages the notion of shared leadership [18]. This implies that leadership is more effective when each individual within an organization takes responsibility for their actions and in turn, this leads to a more cohesive intra-professional network and therefore, a stronger organization.

At the heart of the current NHS reforms are the general practitioners; those who have been made leaders within the Clinical Commissioning Groups and exemplify the ever-changing role of the doctor. Yet they are expected to take on these roles without significant prior training. A survey in 2011 found that 85% of general practitioners felt that they did not have the necessary skills to handle their roles in Clinical Commissioning Groups, of which service commissioning and budget handling is a key responsibility [19, 20]. The lack of doctors at the top level means that individuals with front-line knowledge are not represented enough at the strategy-devising level [21]. By conferring doctors with management and leadership skills, they can be encouraged to take part and benefit the organization as a whole. For example, a large-scale study of 1300 hospitals by McKinsey in conjunction with the London School of Economics highlighted that having clinically qualified managers led to higher performing hospitals as well as better quality patient care [22].

Several senior professionals have been quoted talking about barriers to taking up managerial positions and engaging in formal leadership roles, saying that doctors do not necessarily always jump at the chance of being involved in decision making [23]. The assistant director of leadership at the King’s Fund states that many view it as a ‘burden to be borne, rather than a prize to be won’ [23]. In addition to this, Mark Newbold, a former medically-trained Chief Executive Officer (CEO), said that doctors find the shift in culture daunting since management and medical cultures are fundamentally different [23]. If there was the opportunity for medical students to study management and leadership, they would be equipped with fundamental knowledge such as the organizational structure of the NHS, policy formulation and the economic importance of limited resources and rationing. A medical director on the NHS England area team agrees with this and feels that such training should be a part of medical curriculums [23]. In doing so, students would be primed as future doctors to excel in their leadership roles and once graduated, could then build on the skills they have learnt by utilizing them as junior doctors. This ensures that by the time leadership and managerial challenges are greatest, i. e. at consultant level, skills have been honed to a point whereby doctors can make decisions and lead as effectively as possible.

There has been a drive to make patient-centred care an integral part of modern healthcare delivery. As part of the responsibilities of being a transformational leader, clinicians must effectively engage in both their intra-disciplinary and multi-disciplinary teams. This involves coordinating a variety of professionals and personalities whereby understanding team dynamics and motivation are vital in ensuring an efficient and positive outcome for both the team and patient. Frenk et al. specifically highlight the need for transformative leadership and its value not just within health organizations but also in transforming health systems [24]. Management courses teach such skills and emphasize the importance of organizational behaviour and human resource management. Furthermore, Fitzgerald et al. conducted a study into the status of clinical-managerial hybrids within the NHS and identified that they did not have an appropriate knowledge base and that educational opportunities are limited [25]. This finding was further highlighted in 2011 in Ham et al.’s interviews with 20 of the 22 NHS medically-trained CEOs and found that interviewees’ access to training and development was highly variable [26]. Hence, encouraging the incorporation of leadership and management skills in undergraduate medical curricula will help to standardize training received by medical students.

## How can we solve this problem?

Whilst the importance of management and leadership is stressed within the Medical Leadership Competency Framework, it only gives examples rather than provide formal guidelines on how it can be taught to the undergraduate cohort. The General Medical Council Graduate Outcomes place an emphasis on scientific knowledge and qualities such as communication skills and ethical awareness; however, we must ask ourselves why there is not more emphasis on management and leadership as a key learning outcome [27]. This is somewhat perplexing in a political climate that places an emphasis on evidence-based decisions and practice. Such a view has been reiterated around the world; for example, Brouns et al. conducted a study with Dutch postgraduate medical students who responded stating their desire for more management training within the Dutch medical curriculum [28].

Management is currently only offered as an intercalation degree in two medical schools and this is a poor representation at the undergraduate level, as it represents a mere 6% of medical schools in the UK [29–31]. It additionally requires students to have a prior interest in management to seek the opportunity to study it as an intercalated degree. Moreover, leadership is often taught as a module within the umbrella of management and arguably, management and leadership should be taught hand in hand rather than implying one is more important than the other [32]. Therefore, we suggest that a greater number of medical schools should begin to introduce management and leadership as a combined intercalated degree, thereby increasing their awareness. This is already evident, with Birmingham Medical School offering an Intercalated BSc in Health Management and Leadership for the forthcoming academic year [33].

This suggestion, however, still relies on students actively choosing to intercalate in this degree. In today’s healthcare system, doctors are expected to deliver exceptional medical care as well as manage service delivery, oversee budgets and continuously achieve quality improvement. This is no longer merely a choice for those who are simply interested in this topic, rather it has become a requirement for all doctors. Hence, the ideal solution would be to introduce leadership and management as an integral part of the undergraduate medicine degree. Teaching would ideally be aimed at senior medical students in their clinical years; key topics such as health economics, healthcare strategy as well as organizational behaviour and human resource management would be valuable in preparing students as they develop into ‘Tomorrow’s Doctors’. In addition, Stoller identified six leadership competency domains for physicians that must be targeted in curricula design. These include technical skills and knowledge, industry knowledge, problem-solving skills, emotional intelligence, communication, and a commitment to lifelong learning [34]. Such concepts require a balance between theory and active immersion, with the utilization of various approaches such as group work, case studies and examples. These methods would further enhance teamwork, communication and analytical skills that are essential to doctors.

We acknowledge that there will be challenges to its inception; aspects such as the structure and delivery are of paramount importance and will have to be debated to ensure maximum benefit. Medical schools need to devise curricula that have the ability to meet the challenges of modern day medicine and equip students with valuable skills more than they do at present. This inevitably requires commitment and cooperation from those who are responsible for the delivery of teaching and, as such, these groups must be suitably inspired to instil the same passion within their students. Furthermore, the medical school curriculum in the UK is already quite substantial and this would need to be taken into consideration when incorporating the teaching of management and leadership. Major stakeholders such as governing bodies, deans, lecturers and students must be consulted to develop the curriculum, both in terms of the content, as well as the practicalities of incorporating this into the medical course. Guidance can, and should be taken from the existing intercalations on offer in order to help shape the content of the teaching [29, 30, 33]; however, this will also require commitment from the relevant experts in the field to be available to lecture and organize classes. Whilst the literature we refer to is predominantly UK-based, the introduction of management and leadership into medical school curricula would also be applicable to medical schools worldwide.

Management and leadership are invaluable skills. The NHS is constantly evolving and these skills will undoubtedly be vital in empowering young students and doctors to successfully mould the future NHS. For students, it is crucial that they are able to make astute executive decisions when the time comes. Implementing it for today’s students will enable them as tomorrow’s doctors to tackle the growing challenges of modern healthcare.
